# Acute Liver Failure as the Leading Manifestation of Spontaneous Tumour Lysis Syndrome in a Patient with NonHodgkin Lymphoma: Do Current Diagnostic Criteria of Tumour Lysis Syndrome Need Re-Evaluation?

**DOI:** 10.1155/2019/2358562

**Published:** 2019-12-26

**Authors:** Janja Tarčuković, Lara Valenčić, Željka Polonijo, Ana Fućak, Boban Dangubić, Igor Grubješić

**Affiliations:** ^1^Department of Anaesthesiology and Intensive Care Medicine, Clinical Hospital Centre Rijeka, Krešimirova 42, 51000 Rijeka, Croatia; ^2^Department of Anaesthesiology, Resuscitation, Emergency and Intensive Care Medicine, Faculty of Medicine, University of Rijeka, Braće Branchetta 20, 51000 Rijeka, Croatia

## Abstract

Tumour lysis syndrome (TLS) is a group of pathophysiological processes caused by rapid degradation of tumour cells with subsequent release of intracellular contents into the extracellular space. It is characterized by the development of systemic metabolic disturbances with or without clinical manifestations. The process usually occurs in highly proliferative, large tumours after induction of cytotoxic therapy. Rarely, however, spontaneous TLS can develop, as well as signs of multiorgan failure triggered by an excessive metabolic load and sterile inflammation. The combination of the aforementioned is thus quite unique. Here, we present a 63-year-old male in which spontaneous TLS was accompanied with acute liver failure and delineated underlying nonHodgkin lymphoma. Initial laboratory findings included hyperkalaemia, hyperphosphataemia, hypocalcaemia, uraemia, and increased creatinine levels indicating the onset of TLS with acute kidney injury. Moreover, the patient showed signs of jaundice, coagulopathy, and hepatic encephalopathy. Development of TLS with multiorgan failure prompted rapid initiation of critical care management, including vigorous intravenous fluid therapy, allopurinol treatment, high flow continuous venovenous haemodiafiltration, and commencement of chemotherapy. The case highlights the possibility of TLS as a differential diagnosis in patients presenting with multiorgan failure and the importance of early detection of this potentially challenging and fatal diagnosis.

## 1. Introduction

Tumour lysis syndrome (TLS) is a group of pathophysiological processes that occur when tumour cells start to rapidly degrade [1–3]. The subsequent release of intracellular contents into the extracellular space and blood typically leads to systemic metabolic disturbances with or without clinical manifestations [1–3]. The syndrome usually develops in patients with highly proliferative tumours, a relatively large tumour burden, and a high sensitivity to cytotoxic therapy, such as haematologic malignancies [1–4]. Although rare, spontaneous TLS can occur before the initiation of cytotoxic therapy and accounts for about 1% of all cases of TLS [5–7].

The onset of TLS is characterized by systemic hyperkalaemia, hyperphosphataemia, hyperuricaemia, hypocalcaemia, and uraemia [1–3]. In general, hyperkalaemia and hyperphosphataemia are a result of their release from rapidly lysed tumour cells, hypocalcaemia is related to hyperphosphataemia with precipitation of calcium phosphate in soft tissues, and uric acid represents the end-metabolic product of purines from nucleic acids [2, 7–9]. Since all of these metabolites are eliminated from the body via renal excretion, in the face of large metabolite load from lysed tumour cells, these often precipitate in the renal collecting system [2, 7–10]. Precipitation and crystallization are followed by nephropathy, the inability of the kidneys to excrete solutes and development of acute kidney injury [1–3]. If these derangements progress further, possible complications include acute kidney failure, lethal arrhythmias, neurological complications, and death [1–4, 9–11].

On top of mentioned metabolic products, damaged or stressed tumour cells can release a wide range of mediators called damage-associated molecular patterns (DAMPs), which trigger sterile inflammation [12–15]. As with other metabolic contents, DAMPs from tumour cells can be released spontaneously or during chemotherapy [12, 16]. This may lead to toll-like receptor-mediated inflammasome activation, neutrophil activation, and release of reactive oxygen species, inflammatory mediators, and proteolytic enzymes with subsequent development of systemic inflammatory response syndrome (SIRS) and multiorgan failure [12, 14, 15].

Here, we present a 63-year-old, previously healthy, male in which acute liver failure was the leading manifestation of spontaneous TLS and delineated underlying nonHodgkin lymphoma (NHL).

## 2. Case Presentation

A 63-year-old white male presented at the Emergency Department (ED) with abdominal pain and nausea. Detailed medical history revealed that the patient had suffered from loss of appetite, upper abdominal pain, and fever for two weeks. Apart from mentioned, the patient was previously in good general health and without any chronic therapy.

**Figure 1:**
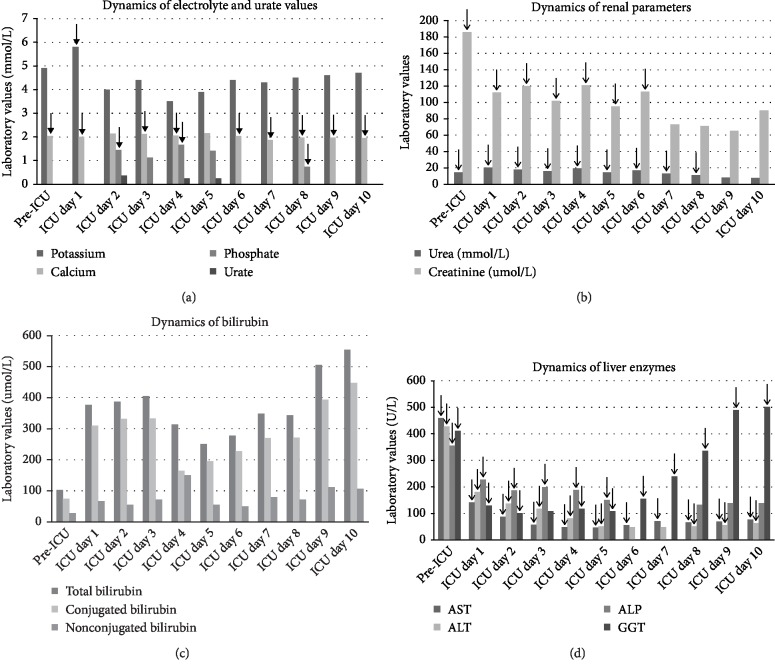
Dynamics of laboratory values during ICU hospitalisation. The potassium and phosphate levels decreased after the initiation of high flow continuous venovenous haemodiafiltration (CVVHDF), while calcium levels started to rise simoultaneously (a). Renal parameters showed decreasing trend after commencement of CVVHDF (b). Both conjugated and nonconjugated bilirubin showed some initial improvement. However, they remained high throughout the course of ICU-treatment (c). After initial improvement, the liver enzymes showed progressively high cholestatic pattern (c, d). Black arrows mark values above laboratory reference range ((a)–(d)).

**Figure 2:**
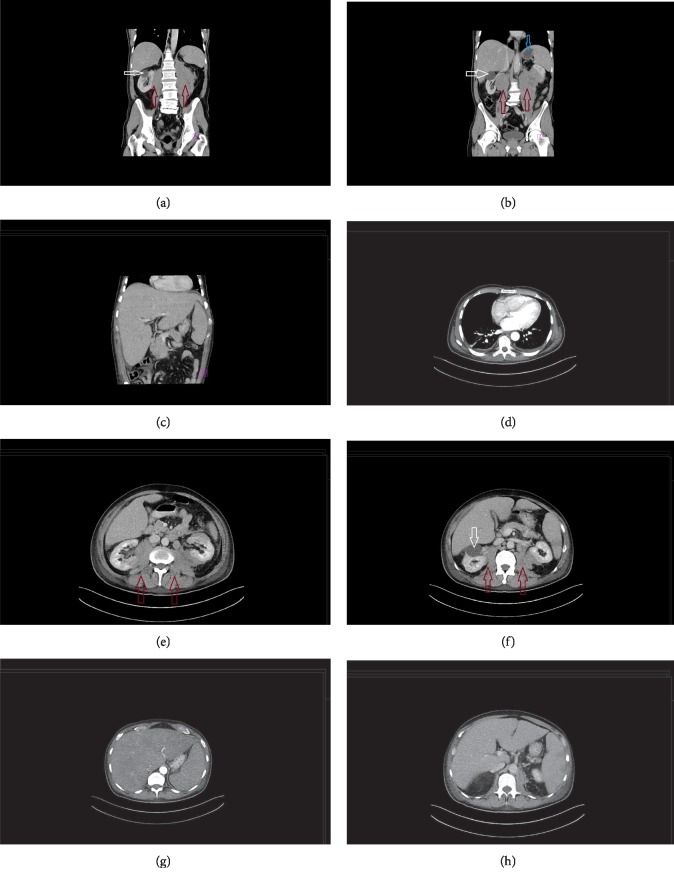
Computed tomography (CT) enhanced with the oral intake of diatriazoate meglumine and diatrizoate sodium contrast solutions. CT scans visualised a retroperitoneal mass of undetermined characteristics with infiltration of both iliopsoas muscles and kidneys, as shown both on sagittal and coronal views (marked with a red arrow in figures (a), (b), (e), and (f)). In the right kidney, a cyst measuring 4.5 cm was seen (marked with a white arrow in sagittal and coronal views in figures (a), (b), and (f)). An infarction zone in the spleen is shown in coronal view (marked with a blue arrow in figure (b)). There were no signs of bile duct dilation (as seen on figures (g) and (h) in sagittal view and (c) in coronal view). Sagittal view of the thorax did not show pathological changes (d). There were no specific signs of obstruction in renal system due to retroperitoneal mass, as well as no infiltration of liver and spleen (figures (a)–(c), and (e)–(h)).

Initial physical examination at the ED showed an enlarged liver and jaundice. Laboratory tests indicated leucocytosis, increased creatinine and urea levels, elevated liver enzymes and hyperbilirubinaemia with cholestatic pattern, and impaired blood coagulation tests (Figure 1 and Table 1, time point: pre-ICU). Radiology investigation included chest X-ray (CXR) and abdominal ultrasound, followed by computed tomography of thorax and abdomen. CXR was within normal limits and excluded acute inflammation or infiltration of the lungs. Abdominal ultrasound showed an enlarged spleen and liver, the latter without focal lesions or dilation of intra- or extrahepatic bile ducts and gall bladder of normal size and wall thickness. Moreover, a retroperitoneal mass of undetermined characteristics was visualized in close proximity to the left kidney, measuring 7 cm in diameter. In the right kidney, a parenchymal cyst was detected, measuring 4.5 cm in diameter. The remainder of the ultrasound examination was within normal limits. Subsequent computed tomography (CT) enhanced with orally administered diatriazoate meglumine and diatrizoate sodium solution revealed a retroperitoneal mass with complete infiltration of both iliopsoas muscles and posteromedial portions of both kidneys, separate mass in the ventral portion of the left kidney and enlarged liver and spleen (Figure 2). No signs of urinary tract dilation were visible. Liver measured 25 cm in craniocaudal diameter and, despite cholestatic pattern in laboratory investigations, showed no signs of obstruction or dilatation in the biliary tract. Spleen measured 15.3 cm and had an infarction zone. Simultaneously with these diagnostic imaging methods, a needle biopsy of the retroperitoneal mass was made, after the correction of coagulopathy with 20 ml/kg of fresh frozen plasma.

After the procedure, clinical state of the patient progressively deteriorated within the next few hours with both clinical and laboratory signs of acute liver failure and acute kidney injury, raising suspicion of TLS. Physical examination using ABCDE approach showed that the patient had patent airway, respiratory rate 22 breaths/minute, SpO_2_ 92–95% with supplemental oxygen (2 L via nasal cannula), normal finding over the lungs on auscultation and percussion, blood pressure of 150/75 mmHg, heart rate of 95/min that was palpable over central and peripheral arteries, capillary refill time <2 seconds, skin warm and sweaty, Glasgow Coma Score measured 13 (E3, V4, M6) with progressively worsening agitation, pupils were equally sized and reactive to light, no lateralisation were noticed, body temperature was 36.9°C, abdomen was above the level of the thorax with palpable liver, painful on deep palpation and intraabdominal pressure measuring 13 mmHg. Laboratory tests at that stage showed profound hyperbilirubinaemia with elevated liver enzymes, hyperammonaemia and coagulopathy (prolonged APTT and PT/INR), together with thrombocytopaenia, increased lactate dehydrogenase (LDH) levels, hyperkalaemia, hyperphosphataemia, hypocalcaemia, and uraemia (Table 1, time point: ICU-day 1), and the patient was admitted to intensive care unit (ICU) with SAPS II score of 52 and predicted in-hospital mortality of 50.7%. Due to the development of severe agitation and hepatic encephalopathy, followed by decreased level of consciousness and inability to properly maintain patent airway, the patient was sedated, an endotracheal tube was placed to protect the airway and lung protective ventilation was started. No cardiac dysrhythmias were recorded on the initial 12-channel ECG or during continuous monitoring via the 3-channel ECG. Orientational echocardiography was made and showed heart of normal size, contractility and valvular function, together with collapsible vena cava inferior and no pericardial effusion. Vigorous treatment with balanced intravenous fluid supplementation was commenced, followed by the initiation of high flow continuous venovenous haemodiafiltration with albumin replacement, broad-spectrum antibiotics and allopurinol therapy. Rasburicase would have been a preferential therapy over allopurinol due to its higher efficiency and the fact that it rapidly reduces existing hyperuricaemia. Unfortunately, in our hospital it was not available at that time. Acid-base and electrolyte disturbances, specifically hypocalcaemia and hyperkalaemia, were corrected in accordance with regular laboratory assessments and with the help of renal replacement therapy (RRT).

When the biopsy results arrived the next day, the induction therapy with methylprednisolone and cyclophosphamide was started (ICU-day 2). In particular, the specimens contained numerous mitoses and apoptotic cells, together with abundant necrotic areas and plain cellular nuclei. The tumour cells had centroblast/immunoblast morphology, immunophenotype that corresponded to peripheral lymphocytes B NHL (Diffuse Large Cell Lymphoma B, DLBCL), double BCL-2/C-MYC protein expression (CD20+/BCL-2+/BCL-6+/MUM-1+/CD10−/CD5−/CD3−) and C-MYC positive in 50% of cells. The constellation of findings consisting of hyperkalaemia, hypocalcaemia, and hyperphosphataemia on ICU-day 1, together with acute kidney injury and abovementioned biopsy findings, lead to the confirmation of previously suspected TLS.

Provided measures and therapy lead to improvement of patient's condition and initiation of triple-drug cytostatic therapy on ICU-day 3. In the following days, patient's laboratory signs showed gradual improvement in electrolyte, urate, and renal function tests, as well as liver enzymes. The patient was weaned from RRT on the ICU-day 5, with subsequent spontaneous diuresis augmented with fractional administration of furosemide and measuring >1 ml/kg/h. On ICU-day 8, the patient was fully awake, cooperative, and weaned from the ventilator. He was transferred to the High Dependency Unit of the Haematology Department on ICU-day 10 fully awake, respiratory and hemodynamically stable with normalization of potassium, calcium, phosphate, ammonium, and urea levels (Table 1, ICU day 10). However, although both conjugated and nonconjugated bilirubin showed some initial improvement, they remained high throughout the course of ICU-treatment and the leading manifestation of the underlying DLBCL (Table 1, time point: ICU-day 3–9). Following ICU, the patient continued haematology treatment and was discharged from the hospital 55 days after the admission.

## 3. Discussion

TLS is potentially lethal oncologic emergency [1, 3, 8, 11]. However, it usually occurs in patients with known underlying disease and after the initiation of cytotoxic therapy [1, 8–10]. In the case presented here, the patient did not have a diagnosed malignancy at the presentation. On contrary, it was a specific laboratory pattern consisting of hyperkalaemia, hypocalcaemia, upper limit values of phosphates, and high LDH levels together with a retroperitoneal mass of undetermined characteristics, in previously healthy patient, which raised suspicion of spontaneous TLS. Symptomatic treatment of acute liver failure and acute kidney injury preceded the definitive diagnosis of DLBCL, after which induction, and then triple-drug cytostatic therapy was started when final diagnosis was made. Taken into account patient's rapid deterioration from full health, laboratory findings, and histology results showing haematological malignancy with high proliferation grade, the diagnosis of TLS was made. In 2004, Cairo and Bishop proposed distinction of TLS depending on whether laboratory changes are followed with the development of clinical symptoms [1, 3]. Laboratory TLS (LTLS) is diagnosed when two or more of the following abnormalities are present in the laboratory tests from three days before to seven days after initiation of chemotherapy: uric acid (UA) ≥476 mmol/L, phosphorus ≥2.1 mmol/L in children and ≥1.45 mmol/L in adults, potassium ≥6.0 mmol/L and calcium ≤1.75 mmol/L (corrected), <0.3 mmol/L (ionized) or 25% increase from baseline for the first three and 25% decrease from baseline for calcium [1, 3]. Presence of LTLS along with increased creatinine levels, seizures, cardiac dysrhythmia, or death constitutes clinical TLS (CTLS) [1]. Following the aforementioned, it is clear that Cairo and Bishop laboratory criteria were only partially met despite the obvious presentation of TLS [1]. We agree with Weeks and Kimple statement that these generally accepted criteria might be insufficient for the diagnosis of spontaneously occurring TLS, given its somewhat different characteristics [6]. In this particular case, the patient was rapidly deteriorating and was promptly admitted to the ICU, where he was closely monitored and received immediate and repetitive treatment of hypocalcaemia, as well as RRT for the rapidly progressing acute kidney injury and correction of hyperkalaemia. Unfortunately, first measurement of urate levels took place after the diagnosis of TLS was made (ICU-day 2), when high flow continuous venovenous haemodiafiltration was already ongoing for several hours. Given the fact that the patient was previously healthy, we can speculate that there was a 25% change in baseline values for laboratory tests needed for confirmation of TLS. However, no previous laboratory findings were available for comparison.

Apart from spontaneous TLS with acute kidney injury, a leading finding in our patient was acute liver failure, presenting as jaundice, pronounced hyperbilirubinaemia with elevated liver enzymes, coagulation disorder, and hepatic encephalopathy. Reviewing the literature, only scarce reports of acute liver failure in a patient with TLS were found, mostly with primary hepatic malignancies and, as such, acute liver failure was never considered a clinical manifestation of TLS [6, 17–20]. In reported cases, TLS usually developed after the initiation of locoregional therapies, chemo- or radiation therapy, when substantial cells degradation occurred [17–19]. Similar observations were made with metastatic involvement of the liver [21]. Given the fact that the underlying diagnosis was a highly proliferative haematological malignancy and that radiology investigations excluded hepatic infiltration and/or cholestasis, a hypothesis was made that acute liver failure resulted from an extreme metabolic load that overwhelmed compensatory mechanisms, together with sterile systemic inflammatory response syndrome (SIRS) triggered by DAMPs from damaged tumour cells [22]. Leucocytosis, increased levels of inflammatory biomarkers and negative microbial cultures contribute to our hypothesis that damaged cells led to sterile systemic inflammatory response syndrome and multiorgan failure. However, pronounced activation of immune response together with the diagnosis of haematological malignancy made the patient susceptible to infection. In order to avoid another insult in already severely compromised patient, broad-spectrum antibiotics were started. Although, as already mentioned, reports of acute liver failure in TLS are scarce, we believe that the primary liver function in metabolic processes makes this organ potentially highly susceptible to injury when faced with extreme metabolic load from damaged cells. Accordingly, we suggest routine investigation of liver function tests when TLS is suspected.

Corroborating the fact that TLS criteria have the potential to be improved since too little attention has been focused on this potentially fatal diagnosis, we can conclude that our patient had SIRS and multiorgan failure due to the underlying haematologic malignancy. We acknowledge the fact that our patient's laboratory findings did not fully meet Cairo and Bishop's criteria for laboratory TLS. However, the patient had clear laboratory pattern consistent with TLS, pronounced SIRS and multiorgan failure. In the absence of other possible causes for this profoundly deranged state, it is reasonable to assume that the DLBCL was the underlying cause. Therefore, we believe that reports on TLS patients' clinical course and different organ involvement should be intensified in order to set new diagnostic guidelines, taking into account the patient's general condition, underlying disease, and course of clinical treatment. In that way, TLS as a life-threatening condition, that can be obscured by the clinical image of multiorgan failure, can be properly recognized.

## Figures and Tables

**Table 1:** 

Laboratory blood tests/measuring unit	Reference values	Pre-ICU	ICU DAY 1	ICU DAY 2	ICU DAY 3	ICU DAY 4	ICU DAY 5	ICU DAY 6	ICU DAY 7	ICU DAY 8	ICU DAY 9	ICU DAY 10
Red blood cells (×10^12^/L)	3.86–5.08	5.21	4.23	4.19	4.74	4.54	4.17	4.08	3.96	3.91	4.05	3.71
Haemoglobin (g/L)	119–157	149	125	123	135	133	121	120	116	117	120	111
Haematocrit (L/L)	0.356–0.470	0.445	0.367	0.358	0.411	0.395	0.361	0.353	0.343	0.342	0.355	0.327
White blood cells (total) (×10^9^/L)	3.4–9.7	10.4	8.3	8.5	10.4	11	10.1	9.5	7	8.4	10.4	10.7
Platelets (×10^9^/L)	158–424	85	61	49	31	58	47	34	47	43	44	58
Serum glucose (mmol/L)	4.4–6.4	5.3	9.2	9.9	11.5	10.1	10.6	13.2	12.4	12.7	8.2	7.3
Urea (mmol/L)	2.8–8.3	14.6	20.1	18.1	16	19.2	14.4	16.7	13.1	11	7.9	7.8
Creatinine (umol/L)	49–90	186	112	120	102	121	95	113	73	71	65	90
Sodium (mmol/L)	137–146	137	151	147	141	140	138	142	141	139	135	135
Potassium (mmol/L)	3.9–5.1	4.9	5.8	4	4.4	3.5	3.9	4.4	4.3	4.5	4.6	4.7
Chloride (mmol/L)	97–108	102	109	108	104	104	102	103	99	99	98	99
Calcium (mmol/L)	2.14–2.53	2.04	2	2.14	2.12	2.05	2.16	2.03	1.86	1.96	1.97	1.95
Phosphate (mmol/L)	0.79–1.42		1.44	1.12	1.66	1.41				0.73		
Total bilirubin (umol/L)	3–20	102	377	387	405	314	250	278	349	343	506	554
Conjugated bilirubin (umol/L)	<5	74	310	332	333	164	195	228	270	271	394	448
Nonconjugated bilirubin (umol/L)		28	67	55	72	150	55	50	79	72	112	106
Urate (umol/L)	182–403			372		258	245					
Aspartate transaminase (AST) (U/L)	11–38	459	141	88	57	48	46	56	71	66	70	77
Alanine aminotransferase (ALT) (U/L)	12–48	428	181	137	117	82	52	48	48	51	56	64
Alkaline phosphatase	50–142	356	228	186	199	188	151			132	139	138
Gamma-glutamyl transferase (GGT) (U/L)	11–55	411	130	101	109	118	108	155	239	336	490	501
Serum amylase (U/L)	40–140	78	60	57	73	74	34			26		12
Lactate dehydrogenase (LDH) (U/L)	<241	786	1109	797	493	441	343			451	468	458
Ammonia (umol/L)	16–60	118.8	62.6				91.1				57.2	
Lactate (mmol/L)	0.5–1.6		4.6	2	2.5	1.5	2.4	2.5	2.6	2.3	1.2	1
Procalcitonin (ug/L)	<0.5—low risk of sepsis, >2— high risk of sepsis		8.69	8.89		8.1			2.43	1.79	1.37	1.09

*Coagulation tests*												
Prothrombin time (PT)	0.70–1.40	0.25	0.37	0.4	0.44	0.43	0.5	0.49	0.52	0.59	0.58	0.59
INR		2.38	1.81	1.69	1.54	1.55	1.42	1.43	1.38	1.29	1.31	1.29
APTT (s)	25.00–40.00	45.01	35.7	35.21	36	42.18	37.84	33.31	31.14	30.9	32	32.3	
Fibrinogen (g/L)	1.80–4.00	1.38	3.16	2.73	2.69	2.38	2.08	2.03	1.98	2	2.2	2.2
